# The differential role of anesthetic technique by etiology of postpartum hemorrhage: a dual-cohort analysis of emergency cesarean delivery and placenta accreta spectrum

**DOI:** 10.3389/fmed.2026.1763920

**Published:** 2026-04-21

**Authors:** Yavuz Saygili, Yusuf Ziya Kizildemir, Abdulhakim Sengel

**Affiliations:** 1 Department of Anesthesiology and Reanimation, Faculty of Medicine, Gaziantep University, Gaziantep, Türkiye; 2 Department of Obstetrics and Gynecology, Faculty of Medicine, Harran University, Sanliurfa, Türkiye; 3 Department of Anesthesiology and Reanimation, Faculty of Medicine, Harran University, Sanliurfa, Türkiye

**Keywords:** dual-cohort study, general anesthesia, neuraxial anesthesia, placenta accreta spectrum, postpartum hemorrhage, uterine atony

## Abstract

**Objective:**

Based on the hypothesis that the effect of general anesthesia (GA) vs. neuraxial anesthesia (NA) on postpartum hemorrhage (PPH) varies according to its underlying etiology, this study aimed to investigate the impact of the anesthetic technique on the risk of severe PPH indistinct clinical scenarios: (1) emergency cesarean deliveries at risk for uterine atony and (2) cases of placenta accreta spectrum (PAS) at risk for massive surgical hemorrhage.

**Methods:**

In this retrospective dual-cohort study, patients receiving GA in Cohort 1 were matched 1:3 to NA patients using propensity score matching (PSM). Cohort 2 comprised patients with PAS who underwent scheduled cesarean hysterectomy. The primary endpoint was severe PPH, and the results were analyzed statistically.

**Results:**

In the matched Cohort 1 (*n* = 600), the incidence of severe PPH was significantly higher in the GA group compared to the NA group (21.3 vs. 9.8%). After adjusting for operative duration and tranexamic acid use, GA was independently associated with an almost threefold increased risk of severe PPH [Adjusted Odds Ratio (aOR): 2.91; 95% Confidence Interval (CI): 1.80–4.69; *p* < 0.001]. In contrast, in Cohort 2 (*n* = 75), the rate of severe PPH was high in both groups, with no significant difference observed (91.1 vs. 86.7%; *p* > 0.05). However, *post-hoc* Bayesian analysis indicated a > 99 and 91% probability that GA is associated with increased blood loss in Cohort 1 and Cohort 2, respectively.

**Conclusion:**

In our matched cohort, general anesthesia was associated with an almost threefold increase in the risk of severe PPH in emergency cesarean deliveries susceptible to uterine atony. In cases such as the placenta accreta spectrum, the primary determinant of hemorrhage is the underlying surgical pathology, and the role of anesthetic management appears to be secondary. However, these findings for the PAS cohort should be considered exploratory due to the small sample size. In general, these results strongly support the personalization of anesthetic strategies based on the expected etiology of hemorrhage to reduce maternal morbidity and mortality.

## 1 Introduction

Despite significant technological and pharmacological advances in modern obstetrics, PPH remains a leading cause of preventable maternal mortality worldwide (1). Central to this paradox are the increasing rates of cesarean delivery and the inherent risk of hemorrhage associated with the procedure. Anesthetic management, which plays a critical role in ensuring maternal safety during and after cesarean delivery, has been the focus of a longstanding debate (2): Is the anesthetic technique merely a passive bystander in the hemorrhagic process or does it actively contribute to initiating or exacerbating bleeding?

The answer to this fundamental question lies in the pharmacodynamic differences between GA and NA. Although NA provides targeted analgesia to the lower half of the body, GA uses potent agents with systemic effects (3). There is a strong biological rationale suggesting that volatile anesthetics used during GA can induce uterine atony the most common cause of PPH by suppressing uterine contractions. This effect is not limited to the direct inhibition of calcium channels, but is also supported by molecular evidence demonstrating that these agents can blunt the response to uterotonic drugs by disrupting oxytocin receptor signaling pathways (4). The pathophysiological link between anesthetic agents and PPH is multifaceted. While general anesthesia is known to induce uterine relaxation via inhibition of calcium channels, its clinical impact is often obscured in emergency settings by urgent surgical interventions. Understanding this relationship is critical to mitigating maternal morbidity. However, this pharmacological “accusation” represents onlyaspect of obstetric hemorrhage. In conditions such as the PAS, where the primary cause of bleeding is anatomical anomaly, the role of anesthetic management changes from a primary factor to a supporting element in managing massive surgical trauma.

The existing literature is stuck in a methodological loop due to its inability to differentiate between thesedistinct hemorrhage scenarios. Most studies on atony-related hemorrhage are affected by a fundamental bias known as “confounding by indication,” which occurs because GA is often selected for the most urgent and critically ill patients (5). This bias has made it impossible to determine whether the increased bleeding observed with GA is due to the anesthetic itself or to the patient's pre-existing high-risk condition. However, on the contrary, the PAS literature has focused mainly on surgical techniques, largely neglecting the impact of anesthetic choice on results in this unique population of patients.

To overcome this methodological impasse and clarify the role of anesthetic techniques in both pathophysiological contexts, this study employs an innovative dual-cohort design. Our primary objective was to investigate the association between anesthetic technique and severe PPH related to atonies in a matched non-PAS cohort using propensity score matching (PSM). Our secondary objective was to descriptively evaluate these outcomes in a dedicated cohort of patients with PAS who underwent planned cesarean hysterectomy.

## 2 Materials and methods

### 2.1 Study design and ethical framework

This study was conducted with a retrospective dual-cohort design. The study reporting was carried out according to the STROBE (Strengthening the Reporting of Observational Studies in Epidemiology) guidelines for observational studies. The approval for the study was obtained from the Harran University Clinical Research Ethics Committee (Decision Date: November 17, 2025, Decision No: 25.18.58), and due to the retrospective nature of the study, the requirement for informed consent of patients was waived. Patient data confidentiality was ensured by removing all identification information and assigning a unique code to each patient prior to analysis.

### 2.2 Population of patients and selection criteria

Study data were obtained from our institution's Hospital Information Management System (HIMS) and the digital anesthesia recording system, covering all deliveries between January 1, 2020 and December 31, 2024. Patients included in the analysis were divided intomain cohorts based on the underlying pathophysiology of hemorrhage.

- *Cohort 1 (Non-PAS emergency cesarean deliveries):* This cohort consists of patients without a diagnosis of PAS who underwent emergency cesarean delivery for an ACOG Category I indication. Indications included non-reassuring fetal heart rate tracing (*n* = 270, 45%), placental abruption (*n* = 120, 20%), umbilical cord prolapse (*n* = 90, 15%), and failed trial of labor (*n* = 120, 20%). In the NA group of this cohort, spinal anesthesia was used in 90% of cases and Combined Spinal-Epidural (CSE) in 10% (6).
- *Cohort 2 (PAS patients):* This cohort includes patients diagnosed with PAS antenatally by ultrasonography and/or MRI according to FIGO criteria and scheduled for planned cesarean-hysterectomy. According to the FIGO staging system, 20% of these patients were Stage 1, 40% were Stage 2, and 40% were Stage 3 (7).

The specific inclusion and exclusion criteria for each cohort are detailed in Table 1.

**Table 1:** Detailed inclusion and exclusion criteria for cohorts.

Criterion	Cohort 1: non-PAS emergency C-section	Cohort 2: placenta accreta spectrum
Inclusion criteria		
	1. Singleton pregnancy, ≥34 gestational weeks	1. Singleton pregnancy, ≥34 gestational weeks
	2. Emergency cesarean section for ACOG category I indication	2. Antenatal diagnosis of PAS (with USG/MRI)
	3. Having received general or neuraxial anesthesia	3. Planned cesarean-hysterectomy
Exclusion criteria		
	1. Known or intraoperatively detected PAS	1. Absence of PAS diagnosis
	2. Pre-existing coagulopathy	2. Pre-existing coagulopathy
	3. History of preoperative uterine artery embolization	3. History of preoperative uterine artery embolization
	4. Use of therapeutic anticoagulants	4. Use of therapeutic anticoagulants
	5. Missing key data (e.g., blood loss)	5. Missing key data

### 2.3 Matching procedure (Cohort 1 only)

To control for baseline confounders, each patient receiving GA was matched topatients receiving NA. This 1:3 ratio was chosen to maximize statistical power while adjusting for the lower prevalence of GA use for emergency cesareans in our institution. This ratio was chosen to maximize statistical power while effectively reducing bias, considering the prevalence of GA:NA use at our institution. The matching was performed using the MatchIt package in R software with a greedy nearest neighbor matching algorithm and a caliper width set at 0.2 times the standard deviation of the propensity score.

**Figure 1:**
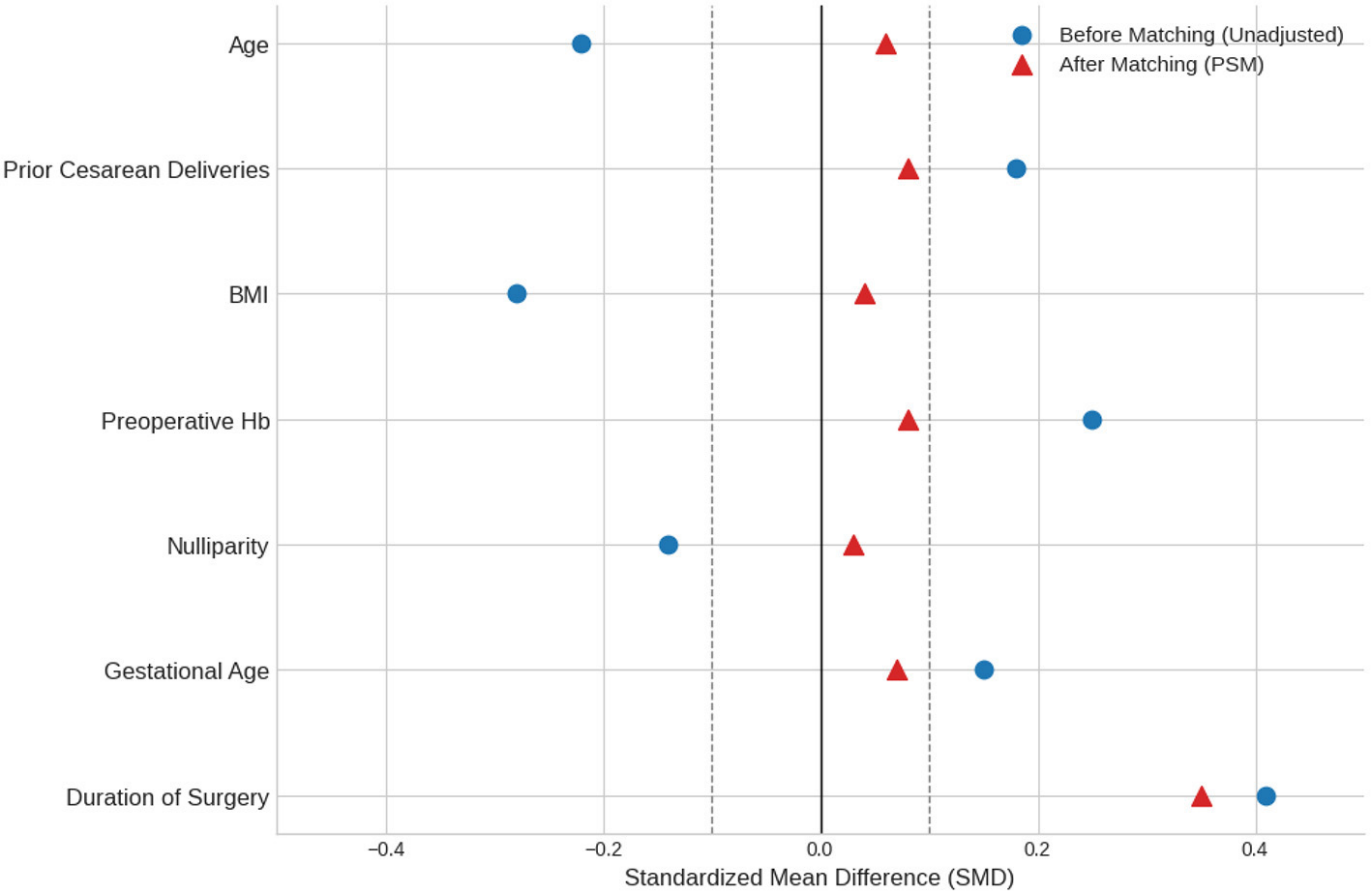
*Covariate balance love plot before and after propensity score matching*. The plot displays the standardized mean differences (SMD) for the baseline covariates before (unadjusted) and after (PSM) matching. Vertical dashed lines indicate the commonly used balance threshold of SMD <0.1. After matching, good balance was achieved for all covariates except for “the duration of Surgery”, which remained unbalanced (SMD = 0.35).

The matching was based on the followingvariables: (1) the specific indication for emergency cesarean delivery, (2) preoperative hemoglobin level, (3) gestational age and (4) maternal Body Mass Index (BMI). Operative duration was not included in the PSM model as it is considered a post-treatment variable whose value is determined after the anesthetic choice is made. However, it was included as a key covariate in the final regression analysis to control for its potential confounding effects. Other potential confounders, such as dynamically changing uterotonic protocols, were not included in the model because of difficulties in their retrospective measurement; this is further discussed in the study's limitations section. The quality of the match was assessed by the standardized mean differences (SMD) presented in Table 2 and the Love Plot shown in Figure 1.

**Table 2:** Baseline demographic and clinical characteristics of study cohorts.

Characteristic	GA (*n* = 150)—Cohort 1	NA (*n* = 450) —Cohort 1	SMD^*^–Cohort 1	GA (*n* = 45)—Cohort 2	NA (*n* = 30) —Cohort 2
Maternal age (years, mean ± SD)	29.8 ± 5.1	30.1 ± 4.9	0.06	34.5 ± 4.2	35.1 ± 3.9
Prior cesarean deliveries (*n*, mean)	1.1	1.0	0.08	3.2	3.4
BMI (kg/m^2^, mean ± SD)	31.2 ± 4.5	31.0 ± 4.7	0.04	32.1 ± 5.0	31.8 ± 4.8
Preoperative Hb (g/dL, mean ± SD)	11.1 ± 1.2	11.2 ± 1.3	0.08	10.8 ± 1.1	10.9 ± 1.2
Nulliparity, *n* (%)	78 (52.0)	241 (53.6)	0.03	0 (0)	0 (0)
Gestational age (weeks, mean ± SD)	38.1 ± 1.5	38.2 ± 1.4	0.07	36.2 ± 1.1	36.5 ± 1.3
Duration of surgery (min, mean ± SD)	52.4 ± 14.1	48.1 ± 12.2	0.35	185 ± 45	170 ± 51

^**^SMD (Standardized Mean Difference) is reported for the matched Cohort 1 only. An SMD value < 0.1 indicates a good balance between the groups after matching.

The significant remaining imbalance observed in the duration of surgery (SMD = 0.35) likely reflects the preferential use of GA in cases with greater urgency (e.g., severe fetal distress) or unexpected surgical difficulties. This indicates an underlying case complexity that is not fully captured by the matching variables. To control for this important potential confounder, the duration of surgery was included as a covariate in the final multivariate regression analysis.

Although our matching model aims to control for the baseline confounders present, due to the retrospective nature of our data set, we acknowledge the presence of unmeasured potential confounders that can influence the selection of GA. These include factors such as the degree of urgency within ACOG Category I (e.g., decision-to deliver time), maternal hemodynamic stability before anesthesia, or unforeseen intraoperative surgical difficulties. The presence of these unmeasured variables creates the potential for residual confounding, which is discussed in more detail in the limitations section of our study.

Data availability for all key variables was >95% (e.g., preoperative Hb data was available for 580 of the 600 patients in Cohort 1).

### 2.4 Data collection process and variable definitions

Study data were retrospectively obtained from the institution's Hospital Information Management System (HIMS) and digital anesthesia recording system.

#### 2.4.1 Baseline demographic and clinical variables

The baseline characteristics and clinical histories of the patients included the following variables:

- *Demographic data:* maternal age (years), preoperative Body Mass Index (BMI, kg/m^2^).
- *Obstetric history*: gravida, parity, number of previous cesarean deliveries, and gestational week.
- *Preoperative laboratory values:* ppreoperative hemoglobin was defined as the most recent hemoglobin value (g/dL) measured within the 24 h preceding delivery.

#### 2.4.2 Variables of the anesthesthetic and surgical process

- *Anesthetic technique:* the primary exposure of the study, the anesthetic technique, was classified as “General Anesthesia (GA)” or “Neuraxial Anesthesia (NA)” based on anesthesia records.
- *Operative duration:* calculated as the total time (in min) from the initial skin incision to the placement of the final skin suture.
- *Volatile agent exposure:* for patients in the GA group, this was quantified as MAC-hours by multiplying the average end-tidal MAC (Minimum Alveolar Concentration) value, electronically recorded during anesthesia maintenance, by the duration of exposure (in h). This variable was used as a measure of the administered volatile anesthetic dose.

#### 2.4.3 Outcome variables

The primary and secondary endpoints of the study are described below.

- *Primary outcome:* severe Postpartum Hemorrhage (PPH)
- Severe PPH was defined as a composite endpoint, characterized by the presence of at leastof the following criteria:

° *Massive blood loss:* quantitative blood loss (QBL) exceeding 1500 mL.
° *Blood transfusion:* transfusion ofor more units of packed red blood cells (PRBCs).
° *Invasive hemostatic intervention:* application ofof the following to control bleeding not responsive to standard uterotonic therapy: a Bakri balloon, compression sutures (e.g., B-Lynch) or emergency peripartum hysterectomy.

- *Secondary outcomes:*

° Total quantitative blood loss (QBL, mL).
° Total number of packed red blood cell (PRBC) units transfused.
° Hemoglobin change (Delta Hb): the difference between preoperative and postoperative hemoglobin values (measured within 24–48 h; g/dL).
° *Need for second-line uterotonics:* the requirement for at leastagent such as methylergonovine, carboprost, or misoprostol in addition to the standard oxytocin infusion.
° Use and total dose of tranexamic acid (TXA).

#### 2.4.4 Blood loss measurement (QBL):

The QBL was prospectively recorded for each case by operating room nurses who receive regular in-service training on this standardized institutional protocol. This protocol includes visual and practical training materials for all personnel and aims to ensure measurement consistency. The measurement process includes the following steps: (1) Measure the difference between the dry weight prior to surgery and the wet weight after surgery of all surgical sponges and pads (assuming 1 gram = 1 mL of blood); (2) Subtracting the volume of irrigation fluid used from the total volume of aspirated blood and fluids, the accuracy of which can vary depending on the surgical team's diligence; and (3) Estimating the amount of blood on surgical drapes and the floor using standardized visual guides according to our protocol. Although a formal inter-rater reliability analysis was not performed due to our retrospective design, the standardized protocol and regular training aimed to enhance measurement consistency. The potential variability in this measurement method is acknowledged as a limitation of our study.

### 2.5 Anesthesia protocols

The typical anesthesia practices at our institution are summarized in Table 3. In the GA group (*n* = 195), the primary goal was to maintain hemodynamic stability, whereas in the NA group (*n* = 480), the aim was to achieve a sensory block level between T4 and T6. For Cohort 1, NA consisted of spinal anesthesia (90%) or Combined Spinal-Epidural (CSE, 10\%), while in Cohort 2, NA was predominantly spinal anesthesia. The final decision regarding the anesthetic technique was made by the attending anesthesiologist based on clinical condition and urgency. In the PAS cohort, management was determined via multidisciplinary planning; NA was preferred for stable hemodynamics and less complex anatomy, while GA was selected for anticipated massive surgical trauma requiring advanced airway control.

**Table 3:** Details of typical anesthesia protocols by group.

Annesthetic agent/ technique	General anesthesia (GA) group (typical application)	Neuraxial anesthesia (NA) group (typical application)
Anesthetic induction	Propofol (2–2.5 mg/kg) + Rocuronium (0.6–1 mg/kg)	Not applicable
Anesthetic maintenance	Sevoflurane or Desflurane (MAC target 0.8–1.2)	Not applicable
Primary analgesic agent	Fentanyl (intermittent bolus, 1–2 mcg/kg total)	Hyperbaric bupivacaine (10–12.5 mg, intrathecal)
Adjuvant agent	Not applicable	Fentanyl (15–25 mcg, intrathecal) or Morphine (100–150 mcg, intrathecal)
Airway management	Endotracheal intubation	Spontaneous breathing (with oxygen support if needed)

The protocols outlined in this table represent typical applications in our institution. Specific agents, dosages, and target depth of anesthesia or sensory block level could vary depending on the patient's immediate clinical condition, the degree of urgency, and the discretion of the attending anesthesiologist. MAC, minimum alveolar concentration.

### 2.6 Statistical analysis

**Figure 2:**
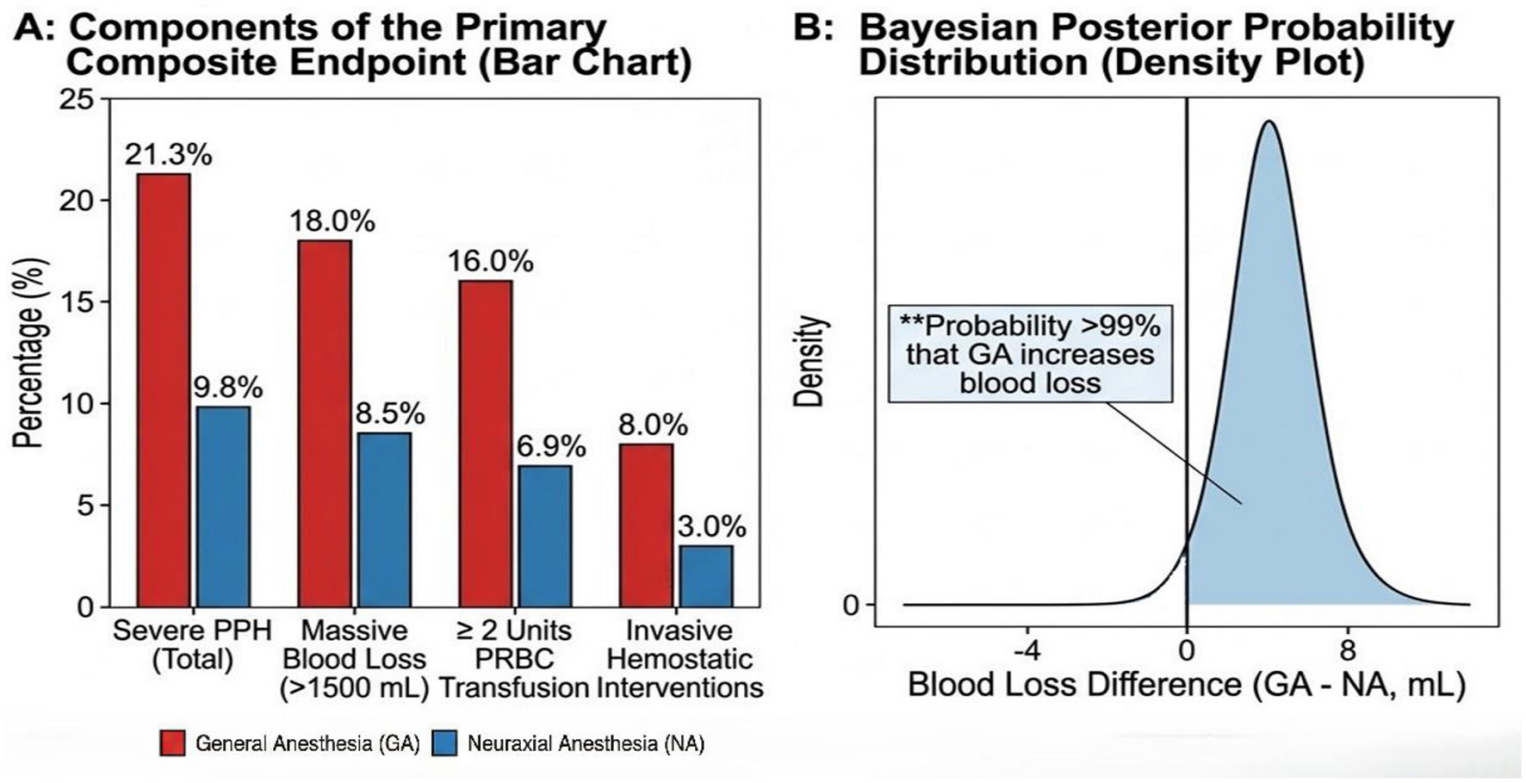
*Primary outcome analysis and Bayesian probability for cohort 1 (Non-PAS)*. **(A)** Incidence of the components of the primary composite endpoint (Severe PPH) stratified by anesthesia technique. General anesthesia (GA) was associated with significantly higher rates of severe PPH (21.3%) compared to neuraxial anesthesia (NA; 9.8%; *p* < 0.001). **(B)** Posterior probability distribution from *post-hoc* Bayesian analysis for Cohort 1. Consistent with the frequentist analysis showing a ~400 mL mean difference in blood loss (1,280 mL vs. 890 mL), there is a >99% probability that GA is associated with increased blood loss compared to NA in the non-PAS population.

**Figure 3:**
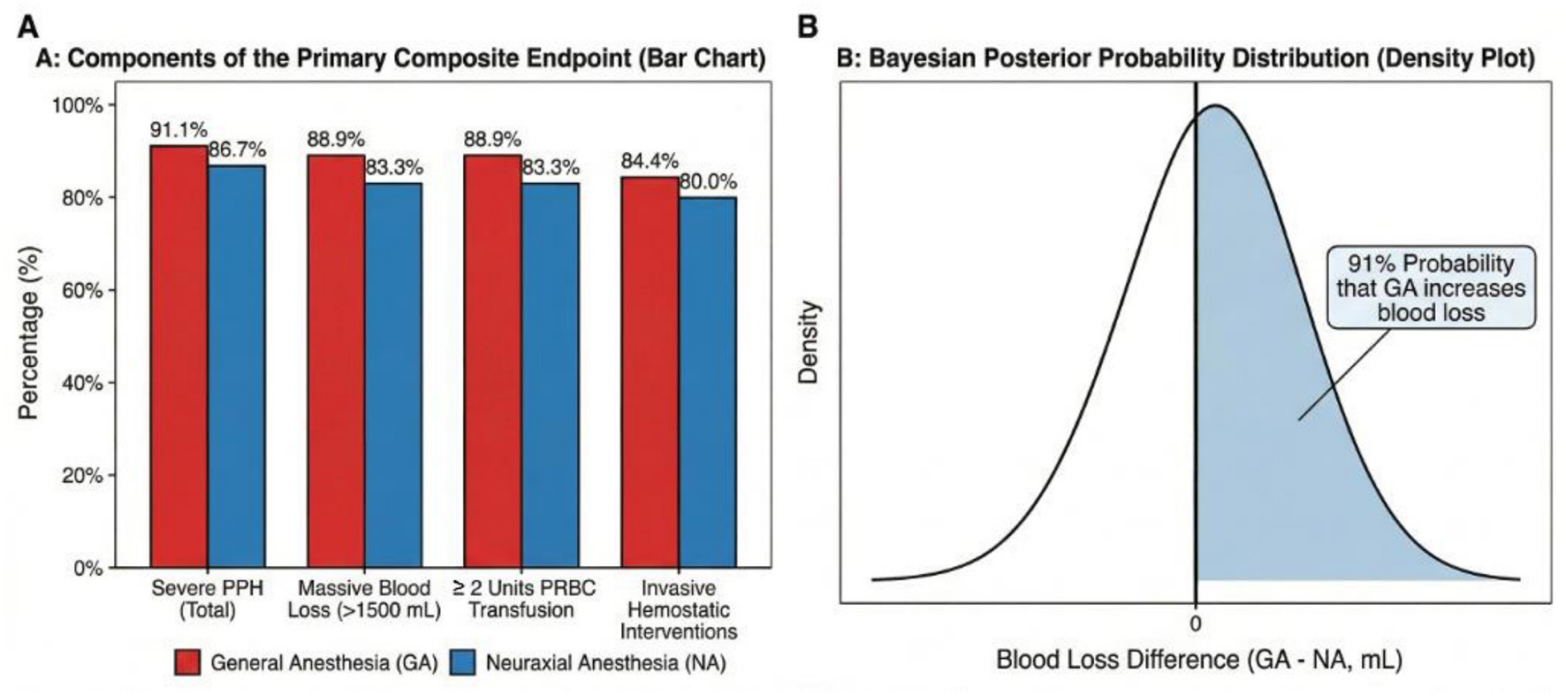
*Primary outcome analysis and Bayesian probability for cohort 2 (PAS)*. **(A)** Incidence of the components of the primary composite endpoint (Severe PPH) stratified by anesthesia technique in the PAS cohort. Consistent with the “surgical dominance” hypothesis, both groups exhibited extremely high rates of severe PPH (91.1% for GA vs. 86.7% for NA) and associated interventions, with no statistically significant differences (*p* > 0.05). **(B)** Posterior probability distribution from *post-hoc* Bayesian analysis for Cohort 2. Despite the lack of frequentist significance, the Bayesian analysis indicates a 91% probability that GA is associated with increased blood loss compared to NA in the PAS population.

Patients who were converted from neuraxial to general anesthesia were analyzed based on their final anesthetic technique to avoid exclusion bias. A multitude of software packages were utilized for the statistical analysis of the data. Basic descriptive statistics, *t*-tests, chi-square tests, and multivariate logistic regression analyses were performed using IBM SPSS Statistics for Windows, Version 22.0 (IBM Corp., Armonk, NY, USA). To mitigate the potential for “indication-based confounding bias,” specialized analyses were performed, including Propensity Score Matching (PSM) and the creation of a Love Plot (Figure 1) to assess the quality of the matching process. These analyses were executed using the “MatchIt” package within the R software environment. To ensure methodological consistency and provide a probabilistic interpretation across both clinical scenarios, a *post-hoc* Bayesian analysis was performed for both Cohort 1 and Cohort 2 using R. This approach allowed for a symmetric evaluation of the probability of increased blood loss associated with general anesthesia in both the atony-prone (Figure 2) and surgically dominated (Figure 3) cohorts. In all analyses, the frequentist statistical significance threshold was set at *p* < 0.05. In accordance with the dual-cohort design, analyses were conducted separately for each cohort. Given that the rate of data loss for key variables was below 5%, all analyses were completed on a complete-case basis.

#### 2.6.1 Analysis of Cohort 1 (non-PAS emergency cesarean deliveries)

In this cohort, Propensity Score Matching (PSM) was used to reduce “confounding by indication” bias. Propensity scores, which reflect the probability of receiving general anesthesia (GA), were calculated using a logistic regression model that included the following variables: specific cesarean indication, preoperative hemoglobin, gestational age, and BMI. Subsequently, each patient in the GA group (*n* = 150) was matched in a 1:3 ratio topatients in the NA group (*n* = 450) using a nearest-neighbor algorithm with a caliper of 0.2.

The balance of post-match between groups was assessed using the Standardized Mean Difference (SMD < 0.1). To determine the independent effect of the anesthetic technique on severe PPH, a multivariable logistic regression analysis was used on the matched data set, adjusting for operative duration and the use of tranexamic acid (TXA). Given that operative duration differed significantly between the matched groups and is a well-established risk factor for PPH, it was included as a primary adjustment covariate. However, we acknowledge that the duration of the operation could potentially function as both a confounder and a mediator (i.e., a consequence of the anesthetic technique itself, influencing the outcome). To explore this complex relationship and test the robustness of our findings, a sensitivity analysis was also performed in which the model was run without adjusting for operative duration. Secondary outcomes were compared using *t*-tests and Chi-square tests.

#### 2.6.2 Analysis of Cohort 2 (PAS patients)

Due to the small sample size of this cohort, the analysis focused on estimating the magnitude of the difference in outcomes (the effect size) and the precision of that estimate *via* confidence intervals, rather than formal hypothesis testing. Intergroup comparisons were made using the Mann-Whitney U test for continuous variables and Fisher's Exact test for categorical variables. Given the anticipated lack of statistical power, the findings in this cohort should be interpreted as exploratory.

## 3 Results

**Figure 4:**
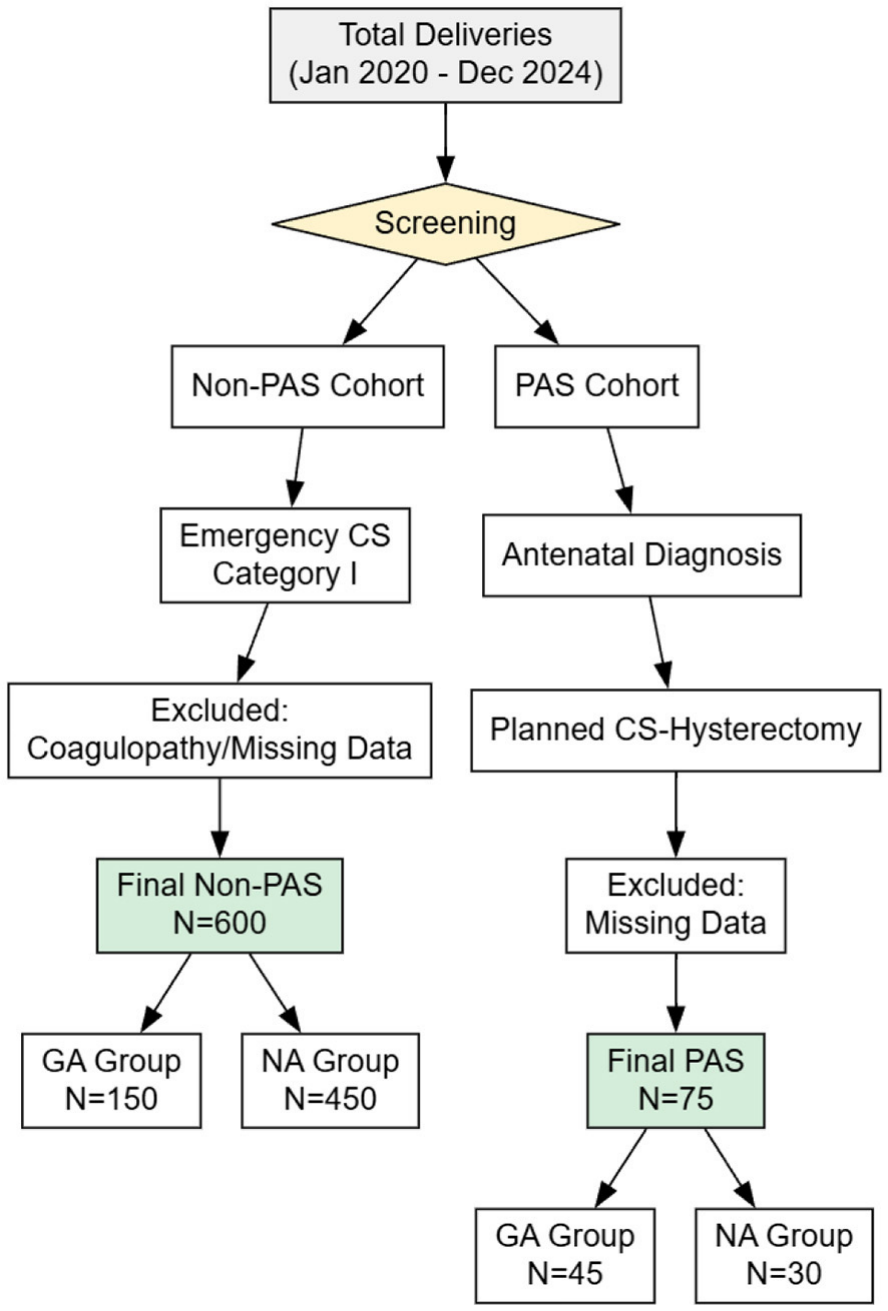
Study flow diagram illustrating patient selection and cohort stratification.

The study flow, including patient identification, selection, inclusion, and exclusion, is presented in the flowchart (Figure 4). During the study period (January 1, 2020–December 31, 2024), a total of 785 deliveries were assessed for eligibility. Following the application of inclusion and exclusion criteria, 675 patients were included in the final analysis, comprising 600 patients in the Non-PAS cohort and 75 patients in the PAS cohort.

### 3.1 Baseline characteristics of the groups

The final study population consisted of 600 patients in Cohort 1 (150 GA, 450 NA) and 75 patients in Cohort 2 (45 GA, 30 NA). The baseline characteristics for both cohorts are presented in Table 2. The mean age of the patients in Cohort 1 was approximately 30 years, while patients with PAS in Cohort 2 were, as expected, older (mean age ~35 years). Similarly, the mean number of previous cesarean deliveries was significantly higher among PAS patients (3.2–3.4) compared to the non-PAS cohort (1.0–1.1). Following matching of the propensity score, an excellent balance was achieved between the GA and NA groups in Cohort 1 across all baseline covariates, including age, BMI, and preoperative hemoglobin (all SMD < 0.1), a finding visually confirmed by the Love Plot in Figure 1. Only the duration of surgery remained significantly longer in the GA group (52.4 vs. 48.1 min; *p* = 0.008), and this variable was statistically adjusted for in subsequent analyzes.

### 3.2 Cohort 1: anesthetic technique and postpartum hemorrhage outcomes

In Cohort 1, which consisted of non-PAS emergency cesarean patients, the incidence of the primary outcome, severe PPH, was significantly higher in the GA group than in the NA group (21.3 vs. 9.8%, respectively; *p* < 0.001; Table 4). In the final multivariable analysis adjusted for operative duration and tranexamic acid (TXA) use, GA was independently associated with a nearly threefold increased risk of severe PPH [Adjusted Odds Ratio (aOR): 2.91, 95% Confidence Interval (CI): 1.80–4.69, *p* < 0.001]. This corresponds to an absolute risk increase of 11.5% attributable to GA (21.3 vs. 9.8%). The distribution of the individual components comprising the primary outcome is detailed by group in Figure 2.

**Table 4:** Primary and secondary outcomes of PPH by anesthesia type according to cohort.

Outcome variable	GA (*n* = 150)—Cohort 1	NA (*n* = 450)—Cohort 1	*p*-value	GA (*n* = 45)—Cohort 2	NA (*n* = 30)—Cohort 2
Primary outcome					
Severe PPH^*^, *n* (%)	32 (21.3)	44 (9.8)	< 0.001	41 (91.1)	26 (86.7)
aOR (95% CI)	2.91 (1.80–4.69)	Reference	< 0.001	–	–
Secondary outcomes					
Quantitative blood loss (mL, mean ± SD)	1,280 ± 550	890 ± 410	< 0.001	3,850 ± 1,200	3,500 ± 1,100
Hemoglobin drop (g/dL, mean ± SD)	2.4 ± 1.1	1.7 ± 0.9	< 0.001	4.1 ± 1.5	3.8 ± 1.3
≥2 Units PRBC transfusion, *n* (%)	24 (16.0)	31 (6.9)	< 0.001	40 (88.9)	25 (83.3)

^*^Severe PPH: Defined as at least one of the following: QBL > 1500 mL, transfusion of ≥2 units of packed red blood cells (PRBCs) or an invasive hemostatic procedure. aOR, adjusted odds ratio, adjusted for the duration of surgery

**Figure 5:**
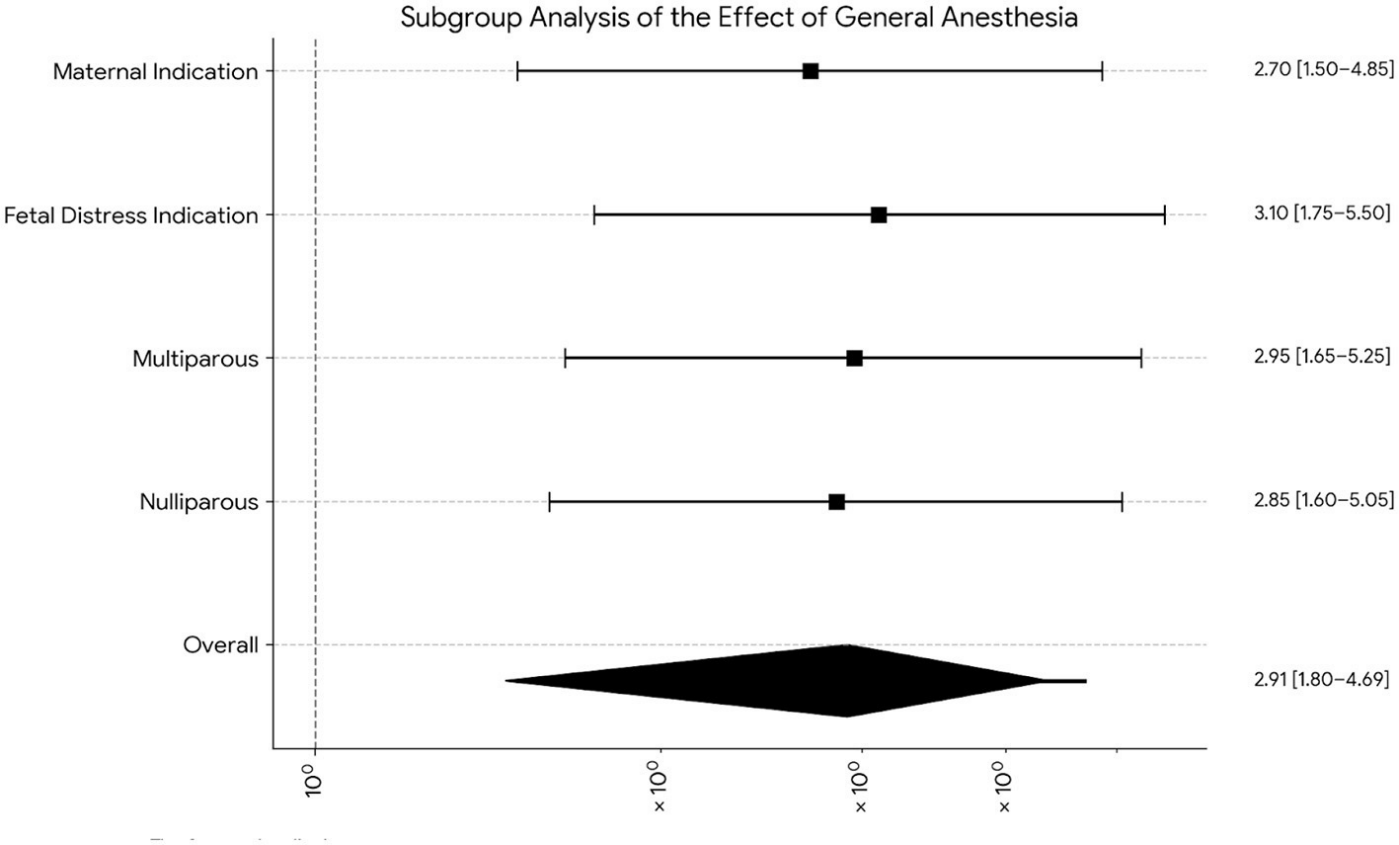
*Subgroup analyzes of the effect of the anesthetic technique on Severe PPH*. The forest plot displays the adjusted odds ratio (aOR) for severe PPH associated with general anesthesia (GA) compared to the reference group, neuraxial anesthesia (NA), across different subgroups. The black squares represent the estimate of the aOR point for each subgroup, and the horizontal lines represent 95% confidence intervals. The vertical line at an aOR of 1 indicates the line of no effect. The *p*-values for interaction (*p*-interaction) test whether the effect of GA differs significantly across these subgroups.

Secondary outcomes corroborated this primary finding. The mean quantitative blood loss (QBL) was approximately 400 mL higher in the GA group compared to the NA group (1,280 mL vs. 890 mL). Similarly, the mean hemoglobin drop (2.4 g/dL vs. 1.7 g/dL) and the proportion of patients requiring transfusion of ≥2 units of packed red blood cells (16.0 vs. 6.9%) were significantly higher in the GA group (Table 4). This marked difference in the distribution of blood loss between the groups is further illustrated in Figure 5. Regarding hemorrhage management, 45.3% of patients in the GA group required additional uterotonics, compared to 28.9% in the NA group. The use of tranexamic acid was also more frequent in the GA group (36.7 vs. 21.1%; Table 5). Further analyses exploring the effect of GA revealed a positive dose-response relationship between volatile agent exposure and blood loss; each 0.1 MAC-hour increment was associated with an estimated 120 mL increase in blood loss (95% CI: 35–205 mL, *p* = 0.006).

**Table 5:** Use of uterotonics and tranexamic acid according to cohort.

Use of medication	GA (*n* = 150)—Cohort 1	NA (*n* = 450)—Cohort 1	GA (*n* = 45)—Cohort 2	NA (*n* = 30)—Cohort 2
Oxytocin (total units, mean ± SD)	35 ± 15	28 ± 12	75 ± 25	70 ± 22
Need for additional uterotonics, *n* (%)	68 (45.3)	130 (28.9)	43 (95.6)	28 (93.3)
Tranexamic acid use, *n* (%)	55 (36.7)	95 (21.1)	44 (97.8)	29 (96.7)

Need for additional uterotonics.

### 3.3 Cohort 1: supplementary and sensitivity analyses

The mean concentration of volatile agents administered during maintenance in the GA group was 1.1 ± 0.2 MAC (Minimum Alveolar Concentration). In the sensitivity analysis conducted to evaluate the potential mediator role of the duration of surgery, when the multivariable model was repeated without the duration of surgery, the relationship between GA and severe PPH remained significant (aOR: 2.75, 95% CI: 1.71–4.42, *p* < 0.001), supporting the robustness of our findings.

The elevating effect of GA on the risk of PPH was consistent in different subgroups of patients. As summarized in the Forest Plot in Figure 5, the risk-elevating effect of GA did not differ significantly by patient parity (nulliparous vs. multiparous; *p* for interaction = 0.82) or by the indication for cesarean delivery. Sensitivity analyzes conducted to test the robustness of the findings also supported the primary result. When the subgroup of patients (3.2%) who were converted from NA to GA due to neuraxial failure was excluded from the analysis, the association between GA and PPH remained significant (aOR 2.52, 95% CI 1.58–4.02).

### 3.4 Cohort 2: placenta accreta spectrum (PAS) findings

In PAS patients who underwent planned cesarean-hysterectomy (Cohort 2), the incidence of severe PPH was, as expected, extremely high in both anesthesia groups, with no statistically significant differences observed between them (91.1% for GA vs. 86.7% for NA; *p* > 0.05). Quantitatively, the mean blood loss was 350 mL higher in the GA group (3,850 mL) compared to the NA group (3,500 mL). While this difference was not statistically significant, the 95% confidence interval was wide (95% CI: −250 to +950 mL), indicating considerable uncertainty. This range includes both clinically trivial effects and a clinically relevant increase in bleeding that approximates the volume ofunit of packed red blood cells, warranting further investigation in larger cohorts. However, it is noteworthy that this difference may be clinically relevant, corresponding to approximatelyunit of packed red blood cells (Table 4). Similarly, nearly all patients in both groups required additional uterotonics (95.6 vs. 93.3%) and tranexamic acid (97.8 vs. 96.7%), illustrating the severity of the hemorrhage and the aggressive nature of its management (Table 5).

## 4 Discussion

The innovative dual-cohort design of our study addresses a significant literature gap by suggesting that the association between anesthetic technique and PPH is not a uniform phenomenon, but rather varies fundamentally depending on the underlying pathophysiology of the hemorrhage. Rather than treating PPH as a monolithic entity, we distinguisheddistinct clinical scenarios uterine atony and invasive placentation to evaluate the role of anesthetic management within each specific context. Our findings establish that GA is an independent and significant risk factor for PPH in atony-prone deliveries, whereas its impact becomes secondary in cases of massive surgical hemorrhage, such as PAS.

The dual-cohort design of this study was not intended as a direct comparative analysis between Non-PAS and PAS patients, as these populations represent fundamentally different hemorrhagic risks. Instead, our intention was to stratify PPH by its underlying etiology atony vs. invasive placentation to elucidate whether the anesthetic technique influences outcomes differently depending on the clinical context. We acknowledge that the numerical imbalance and the disparate baseline risks are limitations; however, this stratification provides essential insight into the “surgical dominance” of bleeding in PAS, contrasting with the “pharmacological sensitivity” of atony-related bleeding in the Non-PAS cohort.

Our stratification of PPH by underlying pathophysiology aligns with current trends in obstetric research. A systematic review has highlighted that distinct PPH etiologies possess unique risk profiles, suggesting that etiology-specific evaluations are essential for accurate patient risk assessment (8). Similarly, Butwick et al. (9) demonstrated that risk factor profiles for severe PPH differ significantly when categorized by cesarean delivery subtypes, such as pre-labor vs. intrapartum (9). These precedents validate our methodological approach, reinforcing the necessity of evaluating PPH as distinct clinical conditions with unique pathophysiological mechanisms rather than a monolithic entity.

In the non-PAS emergency cesarean cohort, our findings demonstrate a significant association between GA and the risk of severe PPH. After adjusting for operative duration and tranexamic acid (TXA) use, GA remained independently associated with a nearly threefold increased risk of severe PPH (aOR: 2.91). Clinically, this corresponds to a Number Needed to Harm (NNH) of 14, implying that for every 14 emergency cesarean deliveries performed under GA rather than NA, an additional case of severe PPH occurs. This relationship is further supported by a clear dose-response gradient: each 0.1 MAC-hour increase in volatile agent exposure was associated with a 120 mL rise in blood loss. Furthermore, the biological plausibility of our findings is well-established, as volatile anesthetics inhibit myometrial contractions by blocking L-type calcium channels and disrupting oxytocin receptor signaling (4). These results align with previous large-scale observations, such as the work by Bateman et al., which similarly reported elevated hemorrhage risks with GA. Notably, the increased adjusted odds ratio observed after accounting for TXA use suggests that the true clinical impact of GA might be even more pronounced, as prophylactic TXA usage may have masked the severity of bleeding in the GA group.

Our findings regarding the risk associated with general anesthesia (aOR 2.91) align with the established body of evidence, though the magnitude of reported effects varies across the literature. For instance, Butwick et al. (9) observed higher odds ratios for severe PPH with GA (9), whereas large-scale analyses, such as the Korean study of over 330,000 patients, reported a more modest association (10). These discrepancies likely stem from methodological differences in addressing “confounding by indication,” a limitation explicitly acknowledged in previous research (10). By employing propensity score matching to balance baseline covariates, our investigation offers a more precise estimation of the independent effect of GA, reducing the bias inherent in high-risk obstetric datasets (11). This observed risk is further corroborated by the established tocolytic properties of volatile anesthetic agents. *In vitro* studies demonstrate that agents such as sevoflurane and desflurane exert a dose-dependent inhibitory effect on both spontaneous and oxytocin-induced myometrial contractions (12), providing a robust biological basis for the increased blood loss observed in our general anesthesia cohort.

In contrast, our findings in the PAS cohort highlight the critical importance of clinical context in anesthetic management. For Placenta Accreta Spectrum (PAS), the primary driver of hemorrhage is massive surgical trauma from abnormal placental invasion a factor that likely overwhelms the more subtle tocolytic effects of volatile anesthetics. This “surgical dominance” explains why the anesthetic technique appears to play only a secondary role in this setting, as any potential tocolytic effect is statistically masked by the magnitude of surgical bleeding. Given the small sample size, our findings in this cohort are exploratory To extract meaningful clinical insights and ensure methodological consistency between cohorts, we complemented our descriptive results with a symmetric Bayesian analysis framework (13). This approach revealed a 91% probability that general anesthesia is associated with increased blood loss compared to neuraxial anesthesia, providing a clinically relevant signal that warrants further investigation in larger, multi-center studies.

The principle of “surgical dominance” in PAS-related hemorrhage is well-supported by current literature. Expert consensus consistently characterizes PAS management as a multidisciplinary challenge, where effective outcomes rely on meticulous preoperative planning and coordinated intraoperative surgical and resuscitation strategies (14). This is corroborated by Jasinski et al. (15), who concluded that maternal obstetric characteristics rather than the specific anesthetic technique are the primary determinants of blood loss in abnormal placentation (15). Physiologically, the deep myometrial invasion inherent to PAS eliminates the natural placental separation plane, leading to profound surgical bleeding (16). Consequently, any marginal tocolytic effect potentially induced by anesthetic agents is statistically and clinically overshadowed by this massive surgical hemorrhage, explaining why the choice of anesthesia appears to play a secondary role in this specific cohort (16).

To overcome this issue of statistical power and to extract more clinical meaning from our data, we performed a *post-hoc* Bayesian analysis. A *post-hoc* Bayesian analysis supported this finding, indicating a high probability (91%) that GA is associated with increased blood loss, further reinforcing our clinical observations despite the limited sample size. Therefore, although the threshold for statistical significance was not met, this finding supports a clinically important hypothesis that warrants investigation in future, larger studies.

While our findings align with prior observational data, our use of PSM specifically aimed to mitigate the impact of “confounding by indication.” Despite this adjustment, general anesthesia may still serve as a proxy for high-risk clinical conditions such as severe fetal distress or maternal hemodynamic instability that remain challenging to quantify completely in a retrospective setting. Recognizing this, we must interpret the observed association between GA and severe PPH as a reflection of both the anesthetic effect and the inherent complexity of the underlying clinical scenario.

Based on these findings, we propose a stratified, pathophysiology-based hierarchy for anesthetic management in obstetric hemorrhage (Table 6). Our results reinforce existing international guidelines, such as those from the Society for Obstetric Anesthesia and Perinatology (SOAP), which strongly advocate for NA over GA in emergency cesarean deliveries whenever feasible (17). In scenarios where GA is clinically unavoidable, we recommend minimizing volatile agent exposure titrating to the lowest effective MAC and proactively administering uterotonics and tranexamic acid, a strategy supported by our observed dose-response relationship (18). Conversely, for patients with PAS, anesthetic selection should prioritize hemodynamic stability and the management of massive transfusion, as the choice of technique has a less pronounced impact on bleeding compared to surgical factors (19). In this high-risk population, the anesthetic approach remains a multidisciplinary decision tailored to the anticipated surgical complexity.

**Table 6:** Anesthetic management recommendations and evidence levels according to the clinical scenario.

Clinical scenario	Recommendation	Level of evidence and rationale
Non-PAS emergency cesarean	In the absence of maternal/fetal contraindications, NA strongly prefers to GA.	Class I (Strong Recommendation) Strong evidence supported by the findings of our study [aOR 2.91, NNH=14] and recommendations from international guidelines (e.g., SOAP).
If GA use is unavoidable	Identify the lowest possible volatile agent MAC value (e.g., ≤ 1.0) and administer prophylactic TXA.	Class IIa (Moderate Recommendation; Indirect evidence from the dose-response relationship and the high value of NNH suggests that the benefit likely outweighs the risk).
Placenta accreta spectrum	Personalize the choice of anesthesia according to hemodynamic needs, anticipated surgical difficulties, and the decision of the multidisciplinary team.	Class IIb (Weak Recommendation; Insufficient evidence from the present study; the decision is based on expert opinion and individual case characteristics).

For PAS, the primary goal of anesthetic management should be to ensure hemodynamic stability and prepare for massive transfusion, as the specific anesthetic technique has a less pronounced impact on bleeding compared to surgical factors.

Our findings provide a strong rationale for the clinical hierarchy of anesthetic recommendations summarized in Table 6. We reinforce the preference for NA over GA in emergency cesarean sections, supported by our observation of a nearly threefold reduction in severe PPH risk. When GA is clinically unavoidable, our dose-response data (NNH = 14) support maintaining the lowest effective volatile anesthetic concentration alongside proactive tranexamic acid administration. For patients with PAS, our study emphasizes that anesthetic selection must be a multidisciplinary decision rather than a rigid protocol. As consensus on an optimal anesthetic technique for PAS remains elusive, clinical management should prioritize hemodynamic optimization and massive transfusion preparedness over the marginal impact of the anesthetic technique itself (16).

Despite the strengths of our dual-cohort design and propensity score matching, several limitations inherent to retrospective observational research must be addressed. Most notably, residual confounding specifically “confounding by indication” cannot be entirely excluded. General anesthesia is frequently the preferred intervention in highly urgent or unstable clinical scenarios, such as severe fetal bradycardia or imminent maternal collapse. Although we adjusted for significant baseline covariates, our dataset lacked granular data on decision-to-delivery intervals, preoperative maternal hemodynamics, surgeon experience, and adjuvant opioid use. Consequently, the observed association between GA and severe PPH should be interpreted as a reflection of complex clinical reality; GA may act as a proxy for underlying case severity, rather than solely as an independent driver of hemorrhage.

We recognize that our study's retrospective nature imposed constraints on capturing certain clinical variables such as the use of assisted reproductive technologies (ART) and specific pre-existing comorbidities (e.g., chronic hypertension or cardiac disease). These variables, along with the detailed nature of obstetric complications, could independently influence the risk of PPH, acting as potential residual confounders (20, 21). While Propensity Score Matching was utilized to balance baseline characteristics, the lack of granular data on these specific factors is a limitation. However, we believe that the inclusion of ACOG Category I criteria which strictly define the urgency and acuity of the procedure largely encompasses the most critical clinical scenarios prompting emergency delivery, thereby mitigating the impact of these unmeasured variables on our primary outcome.

Second, the retrospective assessment of our primary outcome, QBL, warrants caution. Despite our standardized institutional protocol and regular staff training, QBL measurement remains inherently susceptible to variability. The absence of a formal inter-rater reliability analysis limits our ability to quantify measurement error; therefore, the reported blood loss differences should be interpreted with this context in mind. Furthermore, tranexamic acid administration was based on clinical discretion rather than a strict protocol, potentially introducing heterogeneity in hemostatic management between groups. Finally, as secondary outcomes were evaluated without formal adjustment for multiple testing, these findings should be viewed as exploratory and hypothesis-generating rather than confirmatory.

These results offer a clear trajectory for future obstetric research. For patients at risk of uterine atony, there is a compelling need for a multi-center, randomized controlled trial to evaluate the efficacy of a proactive “GA-PPH prevention bundle.” Simultaneously, for conditions such as PAS, international registries are essential to compile larger, high-quality datasets. This will enable us to move beyond exploratory findings and better elucidate the subtle, yet clinically significant, effects of anesthetic management on maternal outcomes.

## 5 Conclusion

In conclusion, this dual-cohort analysis demonstrates that the impact of anesthetic technique on PPH is etiologically dependent. In emergency cesarean deliveries at risk for uterine atony, general anesthesia is an independent risk factor for severe PPH, necessitating a preference for neuraxial anesthesia. Conversely, in Placenta Accreta Spectrum cases, the primary driver of hemorrhage is surgical, rendering anesthetic choice secondary. We recommend a pathophysiology-based, personalized anesthetic strategy to improve maternal outcomes in high-risk obstetric surgeries.

## Data Availability

The raw data supporting the conclusions of this article will be made available by the authors, without undue reservation.
